# New Postcranial Material of the Early Caseid *Casea broilii* Williston, 1910 (Synapsida: Caseidae) with a Review of the Evolution of the Sacrum in Paleozoic Non-Mammalian Synapsids

**DOI:** 10.1371/journal.pone.0115734

**Published:** 2014-12-29

**Authors:** Aaron R. H. LeBlanc, Robert R. Reisz

**Affiliations:** Department of Biology, University of Toronto Mississauga, Mississauga, Ontario, Canada; New York Institute of Technology College of Osteopathic Medicine, United States of America

## Abstract

Here we use the description of a new specimen of the small caseid synapsid *Casea broilii* that preserves the sacral, pelvic and hind limb regions in great detail and in three dimensions, as a unique opportunity to reevaluate the early stages in the evolution of the sacrum in the lineage that led to mammals. We place this new material in the context of sacral evolution in early caseid synapsids and conclude that the transition from two to three sacral vertebrae occurred in small-bodied species, suggesting that it was not an adaptation to heavy weight bearing. Furthermore, we compare descriptions of sacral anatomy among known early synapsids, including caseids, ophiacodontids, edaphosaurids, varanopids, and sphenacodontians and review sacral evolution in early synapsids. Based on the descriptions of new species of caseids, edaphosaurids, and varanopids over the past several decades, it is clear that a sacrum consisting of three vertebrae evolved independently at least four times in synapsids during the Late Carboniferous and Early Permian. Furthermore, similarities in the morphologies of the sacral vertebrae and ribs of these early synapsids lead us to conclude that an anterior caudal vertebra had been incorporated into the sacral series convergently in these groups. Given the repeated acquisition of a three-vertebra sacrum in early synapsids and no apparent link to body size, we argue that this sacral anatomy was related to more efficient terrestrial locomotion than to increased weight bearing.

## Introduction

The evolution of the mammalian vertebral column, especially its degree of regionalization, has attracted attention in molecular, developmental, and evolutionary biology [1]–[3]. These studies have furthered our understanding of the evolution of the mammalian vertebral column from the ancestral condition within Amniota. Non-mammalian amniotes possess a relatively uniform series of rib-bearing dorsal vertebrae posterior to the cervicals that extend to the sacrum, whereas mammaliaform synapsids have developed thoracic and lumbar vertebrae through the loss of the ribs in the posterior dorsal series [3], [4]. The excellent fossil record of extinct synapsids, the amniote lineage that includes mammals, has been crucial in understanding the evolution of the mammalian vertebral column. In a review of axial patterning in extinct amniotes, Müller et al. [2] showed that most reptilian amniote clades possessed a high degree of variability in presacral vertebral counts, whereas this was not the case in synapsid amniotes, including Paleozoic taxa. Müller et al. [2] suggested that this was indicative of developmental constraint in vertebral formulae in Paleozoic synapsids, a feature that is also consistently found in modern mammals. Whereas much of the focus has been on the development and evolution of the presacral portion of the vertebral column in synapsids [1], [3], very few studies have discussed the evolution of the synapsid sacral region. This is in part due to a lack of documentation of the variation in sacral counts for Paleozoic synapsids, thus making it difficult to determine the ancestral condition for major synapsid clades [2]. Such a study would yield important information on the variation in the numbers of sacral vertebrae, but would also provide information regarding terrestrial adaptations in early synapsids, given the functional significance of this part of the axial skeleton.

The sacrum forms the only osseous connection between the axial and appendicular skeletons in tetrapods. The evolution of the sacral region in non-mammalian vertebrates is therefore of great interest because most of the propulsive force in tetrapods is provided by the hind limb musculature and is propagated through the sacral region. Here we describe new postcranial material of the small caseid synapsid *Casea broilii* from the Lower Permian *Cacops* bonebed of Baylor County, Texas, U. S. A., which preserves the sacral region, pelvic girdle, and left hind limb elements in exquisite detail, and place the resulting information within the larger context of early synapsid sacral evolution.

Caseids were one of four early amniote lineages to evolve skeletal adaptations for high-fiber herbivory during the Paleozoic and were also the first to reach massive body sizes, some exceeding 350 kg [5]. Accompanying this evolutionary increase in body size were changes to the postcranial skeleton that are attributed to herbivory and heavy weight-bearing. These include the development of a barrel-shaped trunk to accommodate digestion of plant material, robust limbs, and a strong sacroiliac joint, incorporating three to four sacral vertebrae [6], [7]. Early descriptions of the postcrania of basal caseids indicated that they all possessed three sacral vertebrae [6], [8], which is apparently a derived condition when compared to other equivalent-aged amniotes [9]. The more recent discovery of a small, basal caseid from the Late Carboniferous of Kansas, *Eocasea martini*, shows that the ancestral condition for Caseidae is to possess two sacrals [5]. The question remains whether caseids evolved a sacrum consisting of three vertebrae in response to the drastic increases in body sizes that characterize the group, or in response to a more terrestrial lifestyle. Furthermore, it is unclear if an anterior caudal or a posterior dorsal vertebra was modified into the third sacral [9]. The new material of *Casea broilii* not only furthers our understanding of the postcranial anatomy of the type species for Caseidae and sheds new light on the origin and identity of the third sacral vertebra in caseids, but also allows us to compare patterns of sacral evolution in Paleozoic synapsids.

## Materials and Methods

All permissions were obtained from the relevant institutions (Field Museum of Natural History; Dyke Museum of Natural History, University of Kansas) to loan the material described herein to R. R. Reisz with permissions to study and prepare the specimens. No permits were required for the described study, which complied with all relevant regulations. FMNH UR 2512 (Field Museum of Natural History, Chicago, Illinois) was recovered from the *Cacops* bonebed in Baylor County, Texas, U.S.A. at Indian Creek, near Big Wichita River. All of the material from the *Cacops* bone bed was collected by P. C. Miller of the Walker Museum in 1910 [10]. The site has yielded numerous remains of the small caseid *Casea broilii* and is considered part of the lowermost Vale Formation, or the uppermost Arroyo Formation within the Clear Fork Group, which is Early Permian (Leonardian) in chronostratigraphic age [6]. The material was prepared and photographed at the University of Toronto Mississauga, Canada by D. Scott. The illustrations of FMNH UR 2512 were prepared by N. Wong Ken.

## Results

### Osteological Description

**Figure 1:**
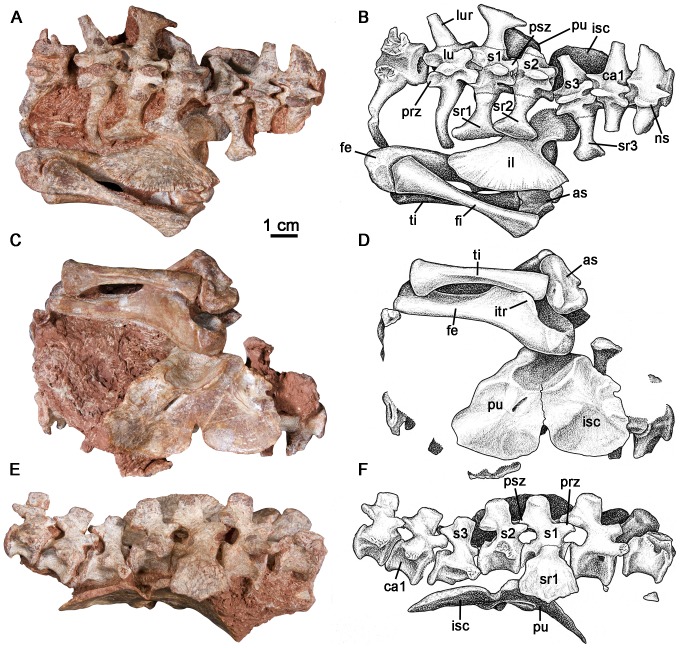
Postcranial remains of *Casea broilii* (FMNH UR 2512). A–B: dorsal view. C–D: ventral view. E–F: right lateral view. *Abbreviations*: as, astragalus; ca1, first caudal vertebra; fe, femur; fi, fibula; lu, lumbar vertebra; lur, lumbar vertebral rib; il, ilium; isc, ischium; itr, internal trochanter; lur, lumbar rib; ns, neural spine; prz, prezygapophysis; psz, postzygapophysis; pu, pubis; s1–3, sacral vertebrae 1–3; sr1–3, scaral ribs 1–3; ti, tibia.

FMNH UR 2512 consists of two posterior dorsal ( =  “lumbar”) vertebrae, three sacrals, the anterior two caudals, a complete left pelvic girdle, a left femur, tibia and fibula, and the left astragalus (Fig. 1) of a small, presumably juvenile caseid. The presence of sutures between the “lumbar” and sacral ribs, as well as a notch along the ventral border of the pelvic girdle and suture extending from this notch to the acetabulum indicate that this animal was not skeletally mature. All of the vertebrae are preserved in or close to their anatomically correct positions along the vertebral column.

Posterior dorsal ribs of caseids are fused or strongly sutured to the transverse processes of the dorsal vertebrae [6]. For this reason the posterior dorsals are referred to as “lumbars” in caseids [6]. The anterior preserved “lumbar” vertebra of FMNH UR 2512 is broken at the neural arch and preserves only the centrum and the left rib. The centrum is shallowly amphicoelous and the anterior and posterior rims of the centra form rounded lips. The ventral surface of the centrum is smooth and rounded. The “lumbar” rib is strongly sutured to the transverse process of the vertebra as it is in other caseids [6]. There is a thin, interdigitating suture between the transverse process of the vertebra and the capitulum and tubercle of the “lumbar” rib. The rib projects laterally from the vertebral body and gently bows ventrally, which would have created a barrel shape to the posterior trunk region. The second “lumbar” vertebra is complete and has a low, rounded neural spine (Fig. 1). The prezygapophyses project anteriorly and the articular facets are slightly concave upward. The postzygapophyses are preserved in articulation with the prezygapophyses of the first sacral vertebra in the horizontal plane. In dorsal view, the “lumbar” rib projects anterolaterally and the distal end curves slightly posteriorly around the anterior margin of the expanded first sacral rib (Fig. 1A, 1B).

Three sacral vertebrae form the sacroiliac joint in this specimen (Fig. 1). All of the neural spines are anteroposteriorly broad and dorsoventrally short. Contrary to Williston's [10] description of *Casea*, the ribs of the sacral vertebrae decrease in mediolateral length posteriorly so that the sacrum tapers posteriorly in dorsal view. There is a faint interdigitating suture between all of the sacral ribs and the transverse processes of the vertebrae. The first sacral vertebra is the largest of the series, with well-developed prezygapophyses that articulate with the posteriormost “lumbar” vertebra. As in other caseids, and in Paleozoic amniotes in general [11], the prezygapophyses of the first sacral are significantly larger and extend farther laterally than the postzygapophyses, which articulate with equally small prezygapophyses of the second sacral and are positioned close to the midline. The first sacral ribs are the largest in the series, are anteroposteriorly narrow, and have dorsoventrally expanded connections of the tubercula to the transverse processes of the vertebra. The proximal end of each rib is not only expanded ventrally, but also bears a small opening that separates the rib tuberculum from the capitulum. The ribs extend laterally from the vertebra and are greatly expanded distally. The distal ends are inflected at approximately 90° such that the dorsal surfaces of the ribs face laterally and would have contacted the ilia (Fig. 1). The lateral surface of the distal expansion is flat and would have contacted the dorsomedial surface of the ilium in a flat buttress. The anterior margin of the distal expansion of the rib is gently convex, whereas the posterior margin, which had a contact with the distal end of the second sacral rib, is concave. The second sacral vertebra has smaller postzygapophyses than those of the first sacral and has a smaller sacral rib. The distal end of the second sacral rib is also inflected at approximately a right angle. The anterior margin of the distal end overlaps the posterior end of the first sacral rib to form a flat articular surface with the sacroiliac joint (Fig. 1A). The posterior end of the rib would have also overlapped the third sacral rib posteriorly, based on the shape of the posterior margin of the distal end of the second sacral rib. The third sacral vertebra has become disarticulated from the anterior two sacrals, but is clearly a part of the sacral series based on the distal expansion of the preserved left rib. The third sacral vertebra has the smallest rib and the smallest distal expansion. This rib curves ventrally at slightly less than 90° and the distal surface is round in lateral view.

Only the two anteriormost caudal vertebrae are preserved. The neural spine of the anterior caudal is short and stout and is similar in dimensions to the neural spines of the sacrals. The prezygapohpyseal facets face dorsally and the postzygapophyses are slightly inclined from the horizontal plane. The transverse processes of the first caudal are slightly wider anteroposteriorly than those of the sacral vertebrae. The prezygapophyses of the second caudal are more steeply inclined medially. The transverse processes are also wider than those of the first caudal vertebra. There are no haemal arches associated with the first two caudal vertebrae.

The left pelvic girdle is preserved near anatomical position with respect to the sacral region. The girdle elements are all completely fused, except for the presence of a notch at the ventral midline of the ischium and pubis and a suture that extends from this notch to the ventral portion of the acetabulum (Fig. 1C, 1D). The ilium is a fan-shaped element with greatly expanded anterior and posterior processes, allowing for confident assignment of this material to *Casea broilii* following the descriptions of Williston [10] and Olson [6]. The medial surface of the iliac blade is flat and smooth with minor striations along the dorsal margin, another diagnostic feature for the genus [6], [10] (Fig. 1A, 1B). The ilium is constricted anteroposteriorly, forming a neck directly above the acetabulum, and then abruptly expands anteroposteriorly into a fan-like blade. Although mostly covered by the proximal head of the femur, the dorsal border of the acetabulum bears a raised lip, which is also seen in *Cotylorhynchus romeri* [12]. A ridge formed by the ilium and pubis brackets the anterior boundary of the acetabulum. No clear sutures between the ilium and the ischium or pubis are visible. The pubis is broad and plate-like, the anterior margin of which bears a small, rounded pubic tubercle. The obturator foramen is elongate and nearly reaches the anteroventral border of the acetabulum. The posteroventral portion of the pubis and the anteroventral portion of the ischium form a thin sheet of bone below the acetabulum.

The femur is exposed in ventral view and is preserved in close association with the pelvic girdle and the distal end is still associated with the tibia and fibula (Fig. 1). The shaft of the femur is comparatively long and slender, much more so than in most other caseids [6], and is similar in proportions to that of *Eocasea*. The proximal articular surface is smooth. From the proximal, medial corner of the bone a thin, low ridge extends distally from the articular surface to a large, crest-like internal trochanter. The internal trochanter projects straight posteriorly and borders a broad, shallow intertrochanteric fossa. In *Cotylorhynchus* and other larger caseids this fossa is much deeper [11]. A low ridge extends distally from the internal trochanter almost to the medial surface of the lateral condyle, which is typical of caseids [6], [11], [12]. There is no expansion along this low ridge for a fourth trochanter. At the distal end of the bone the lateral condyle extends further distally than the medial condyle.

The tibia and fibula are still partially articulated with the femur proximally and the astragalus distally (Fig. 1). The tibial head is only moderately expanded, being slightly wider than the distal end, like in *Eocasea*. In more derived caseids, the tibial head is greatly expanded [6]. The lateral and medial articulations with the femur are separated by a groove along the anterior surface of the bone. The cnemial crest extends nearly along the entire length of the medial surface of the tibial shaft (Fig. 1C, 1D). The fibula is long and slender and has only slightly expanded proximal and distal ends. The bone is twisted slightly so that the proximal and distal articular surfaces are in slightly different planes. Only a small portion of the distal head of the fibula articulated with the astragalus.

**Figure 2:**
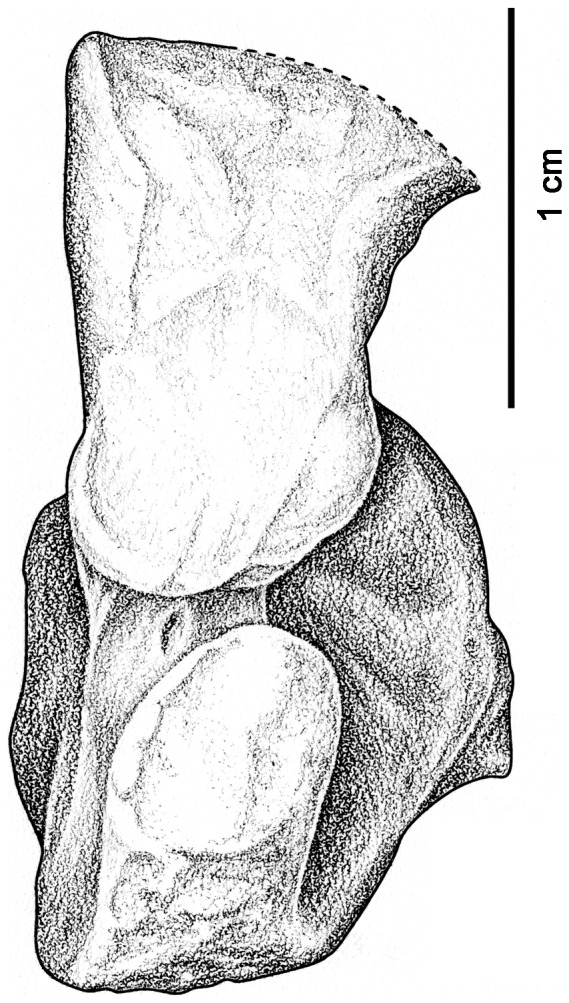
Preserved left astragalus of *Casea broilii* (FMNH UR 2512) in lateral view. Note the presence of a groove for the perforating artery that passes across the entire facet for the calcaneum. Anterior is to the right.

The astragalus is preserved in articulation with the tibia and is only partially disarticulated from the fibula (Fig. 1A–1D). Contrary to the descriptions by Romer and Price [8] and Olson [6], the astragalus of *Casea broilii* is an elongate element in which the articulation for the fibula is separated from the articulation with the tibia by a long neck (Fig. 1C, 1D). This difference in interpretation of the anatomy of the astragalus may have been the result of misidentification of a partial, isolated foot, (UC 657) as belonging to *Casea.* The articulation of this astragalus to the pelvic girdle and hind limb of *Casea* in FMNH UR 2512, raises the possibility that the former specimen represents a different taxon. The facet for the fibula in FMNH UR 2512 is flat, as it is in other caseids [12]. The lateral facet for the calcaneum is also flat and is only interrupted by a groove for the perforating artery. The lateral view of the astragalus of FMNH UR 2512 clearly shows that the perforating artery was equally accommodated by the astragalus and calcaneum throughout, unlike the condition in varanopids (Fig. 2) [13].

## Discussion

### New Insights Into the Postcranial Anatomy of *Casea broilii*

This specimen can be confidently assigned to Caseidae based on the absence of a ventral midline keel on the posterior presacral centra [6], [11], the presence of a well-developed internal trochanter on the femur, and an adductor crest that extends diagonally across the femoral shaft almost to the lateral condyle of the femur [12]. Furthermore, this partial skeleton can be attributed to the genus *Casea* based on the presence of a greatly expanded, fan-shaped ilium with a flat contact to the sacrum [6]. Specifically, this material can be assigned to *C. broilii* based on the lack of any expansion of the proximal tibial head.

**Figure 3:**
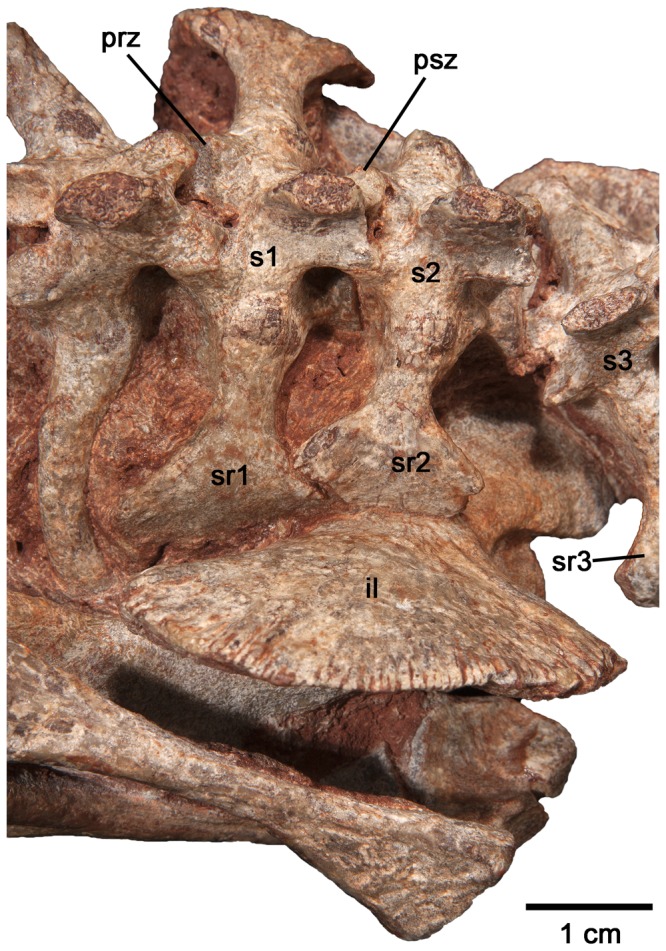
Close-up of the sacral region of *Casea broilii* (FMNH UR 2512). Note the overlap of the second sacral rib over the first sacral rib. *Abbreviations*: prz, prezygapophysis of first sacral vertebra; psz, postzygapophysis of first sacral vertebra; s1–3, sacral vertebrae 1–3; sr1–3, scaral ribs 1–3.

Although it is a reasonably well-known taxon, the new specimen is better preserved and prepared than those used in previous descriptions, the original specimens having been prepared rather crudely before the advent of modern preparation tools. Thus, the newly prepared material permits some corrections to the interpretation of the postcranial anatomy of *C. broilii*. Williston [10] noted that the first two sacral ribs were the same size and that the ilium featured a greatly expanded anterior process and only a moderately expanded posterior one. It is clear from this material that the first sacral ribs are the largest in the series and that the sacral ribs decrease steadily in size posteriorly (Fig. 1). Furthermore, the anterior expansion of the iliac blade is equally matched by an elongate posterior process, which produces the posterior half of the fan shape of the iliac blade. Although Romer and Price [8] and Olson [6] also noted that the distally expanded sacral ribs did not contact each other, this material clearly shows that this interpretation for *C. broilii* is incorrect. The sacral ribs form a single, continuous contact with the ilium which is formed by the overlap of sacral ribs one and two, as well as between ribs two and three (Figs. 1, 3).

The anatomy of the astragalus described here is consistent with those of other caseids, suggesting that the foot previously described as belonging to *Casea broilii* (UC 657) [6] may actually belong to another taxon. This is a reasonable interpretation because the proportions of the pes, as described, appear to be too gracile for a caseid, including slender terminal phalanges. In contrast, all other known caseids, including the small basal taxon *Eocasea*, possess relatively short, broad pedal elements [5].

### The Evolution of the Sacral Vertebrae in Caseidae

**Figure 4:**
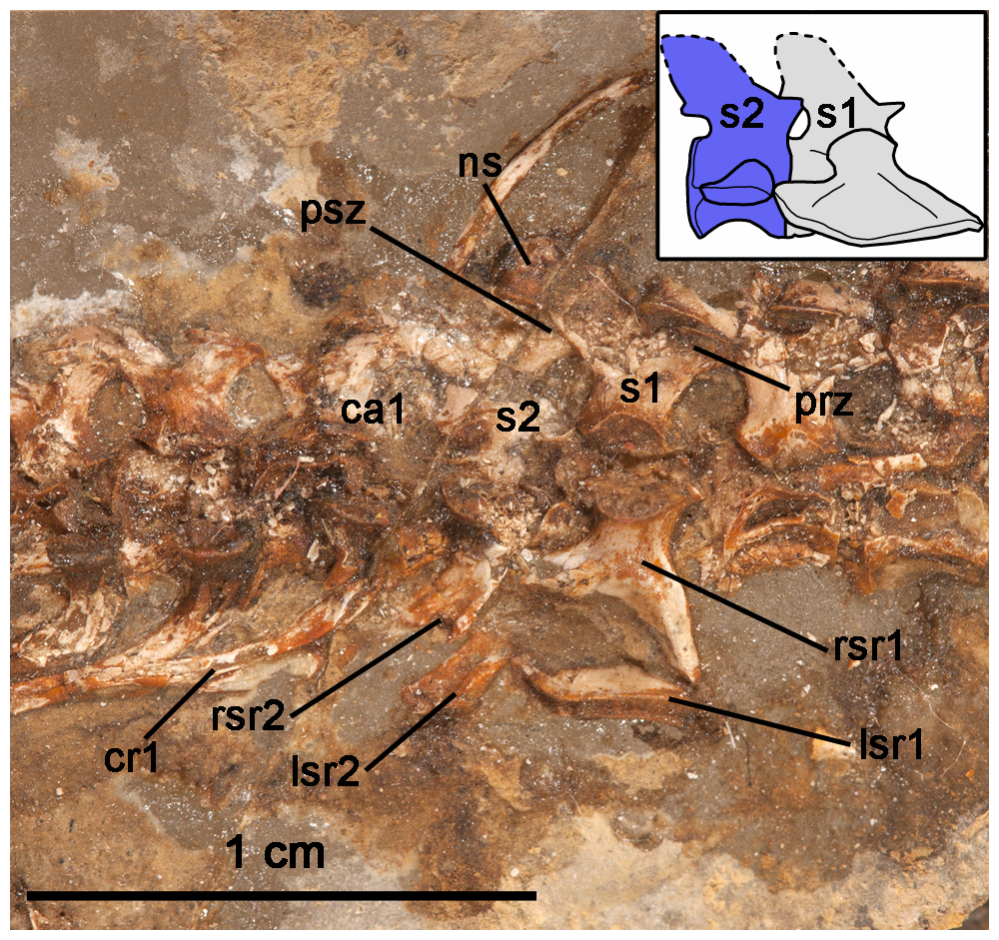
Close-up and reconstruction (inset) of the sacral region of *Eocasea martini* (KUVP 9616b). *Abbreviations*: ca1, first caudal vertebra; cr1, first caudal rib; lsr1–2, left sacral ribs 1–2; ns, neural spine; prz, prezygapophysis of first sacral vertebra; psz, postzygapophysis of first sacral vertebra; rsr1–2, right sacral ribs 1–2; s1–2, sacral vertebrae 1–2.

**Figure 5:**
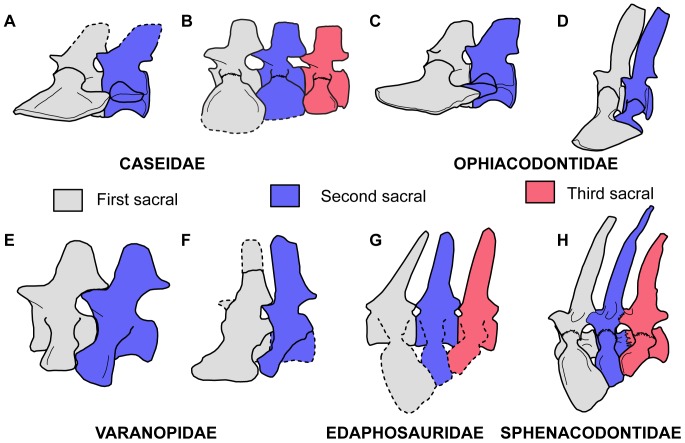
Reconstructions of sacral vertebrae of basal synapsids examined in this paper and hypothesized homologous sacral positions. A: reconstruction of sacral vertebrae of *Eocasea martini* based on the holotype (KUVP 9616b). B: reconstruction of sacral vertebrae of *Casea broilii* based on FMNH UR 2512. C: reconstruction of sacral vertebrae of *Varanosaurus acutirostris* modified from Sumida [19]. D: reconstruction of sacral vertebrae of *Ophiacodon retroversus* from Romer and Price [8]. E: reconstruction of the sacral vertebrae of the basal varanopid *Archaeovenator hamiltonensis* based on the holotype (KUVP 12483). F: reconstruction of the sacral vertebrae of *Varanodon agilis* based on FMNH UR 986. G: reconstruction of the sacral vertebrae of *Edaphosaurus boanerges* based on the description of MCZ 1372 from Romer and Price [8] and ROM 7985. H: reconstruction of the sacral vertebrae of *Dimetrodon limbatus*, modified from Romer and Price [8]. Dashed lines indicate portions of the skeleton that were either extrapolated from the literature or were damaged on the specimens.

New discoveries, anatomical descriptions, and phylogenetic analyses of caseids from North America [5], [14] and Europe [11] permit a discussion of how the sacral attachment has evolved in caseids from the ancestral pattern in basal synapsids. Derived caseids were some of the largest Paleozoic tetrapods, with *Cotylorhynchus*, *Angelosaurus*, and *Ennatosaurus* attaining body masses greater than 350 kg and lengths of over 3 meters ([5]: Appendix S1). Such large body masses would have imposed considerable stresses on the axial and appendicular skeletons and are reflected in adaptations to heavy weight bearing in some of these regions. For example, the limb bones of large caseids are characteristically short and broad and the sacral ribs are strongly attached to the pelvic girdles via rugose articulating surfaces [6], [8], [15]. *Angelosaurus* from the Permian of Texas shows further adaptations to supporting a more massive trunk in the presence of a fourth sacral vertebra, the only caseid to do so [6]. Interestingly, the most dramatic changes to the axial and appendicular skeletons of caseids did not occur in conjunction with the appearance of these heavier forms, but can be traced across the transition to herbivory in much earlier and smaller caseids. Using the phylogeny of Reisz and Fröbisch [5] and the holotypic material of *Eocasea martini* (KUVP 9616b, Dyke Museum of Natural History, University of Kansas), it is now possible to examine the evolution of the sacrum in basal caseids. The most basal and geologically oldest member of Caseidae, the recently described *E. martini*, best approximates the ancestral condition for sacral and pelvic girdle anatomy in caseids [5] (Fig. 4). *E. martini* is unique among caseids in possessing two sacral vertebrae, with the anterior sacral being larger than the posterior vertebra and having significantly larger distal expansions of the ribs for contact with the ilia than the second pair of sacral ribs [5]. The second pair of sacral ribs is short and dorsoventrally flat. The two pairs of sacral ribs also do not contact each other distally, unlike the condition in *Casea broilii* (Figs. 1, 3). Furthermore, the ilium of *E. martini* possesses only a moderate anterior expansion and a slightly larger posterior process. Unfortunately, there is no described postcranial material of the Early Permian caseid *Oromycter dolesorum*, making *C. broilii* the closest relative to *Eocasea martini* with preserved sacral and pelvic girdle elements. *C. broilii* had an estimated adult weight of 21 kg [5], much smaller than the largest caseids, and differs from *E. martini* in the addition of the third sacral vertebra and the anteroposterior expansion of the iliac blade to accommodate an expanded sacral series. Furthermore, the sacroiliac joint in *C. broilii* is formed by three sacral ribs, whereas the joint is formed primarily by the first sacral rib in *E. martini* and most other basal amniotes [8], [9]. We hypothesize that the first caudal vertebra had been incorporated into the sacral series as a third sacral vertebra in *C. broilii*. This hypothesis is supported by parsimony, based on the size and shape differences between the sacrals on the one hand, and the posterior dorsal vertebrae and their ribs, and the anterior caudal vertebrae and their ribs, on the other. The first pair of sacral ribs in *C. broilii* could be homologous to the posteriormost lumbar vertebra and ribs in *Eocasea martini*, similar to some extant mammals which can incorporate lumbar vertebrae into the sacral series [3], but this is unlikely to be the case in *C. broilii*, because morphological and size similarities between the sacrals and the anterior caudals is much greater than with the posterior dorsals [16]. Furthermore, as a landmark of the first sacral vertebra in other amniotes [11], [17], the anteriormost sacral in *C. broilii* possesses small postzygapophyses that are located close to the midline, compared to the prezygapophyses that are more widely separated from the midline (Fig. 3), suggesting that this anterior sacral is homologous with the first sacral in other amniotes. Given the size and morphology of the posterior sacral vertebra and rib, its similarity to the anterior caudal and its ribs, it is significantly more parsimonious to hypothesize that the third sacral of *Casea broilii* is homologous to the first caudal of *Eocasea martini*, and was modified to contact the posterior expansion of the iliac blade and the posterior end of the second pair of sacral ribs (Fig. 5A, 5B).

In addition to a third sacral vertebra, the sacral ribs of *C. broilii* are strongly sutured to the transverse processes, whereas the ribs form a flat plane of articulation with the transverse processes of the sacrals in *E. martini* (Figs. 4, 5A). These comparisons indicate that caseids evolved a sacrum consisting of three vertebrae very early in their evolutionary history and this transition from two to three sacrals occurred in relatively small caseids. Furthermore, three sacral vertebrae remained the stable condition for caseids, despite drastic increases in body size, with the exception of *Angelosaurus* [6].

### The Evolution of the Sacral Region in Early Synapsids

Caseids were not the only Paleozoic synapsids to evolve a sacroiliac joint that incorporated three pairs of sacral ribs. Phylogenetic analyses of Paleozoic synapsids provide compelling evidence that a sacral region consisting of three vertebrae evolved multiple times within Synapsida, and inherited in Therapsida. A brief review of the postcrania in key specimens of ophiacodontids, varanopids, edaphosaurids, and sphenacodontians provides a more complex picture of sacral evolution in these major lineages than previously thought [18], and new insights into how the third sacral vertebra was incorporated into the ancestral two-vertebra sacrum in some of these clades. Furthermore, a better understanding of the timing of the acquisition of an expanded sacral series may shed light on the selective forces that promoted these convergent morphologies.

Unlike the other Paleozoic synapsid clades, all ophiacodontids retain the ancestral arrangements of the sacral vertebrae. In *Varanosaurus acutirostris* and *Ophiacodon retroversus*, the anterior pair of sacral ribs is significantly larger than the second pair [8], [19] (Fig. 5C, 5D). The large anterior pair of ribs forms the contact between the sacrum and the ilium and the second pair extends anterolaterally to the posterior surface of the first pair of ribs. This configuration allows for the first pair of sacral ribs to form the attachment of the axial skeleton to the pelvis, whereas the second pair of ribs provided support for this joint [9]. The similarities between the sacral regions of *V. acutirostris* and other basal amniotes (see Reisz [16]) suggests that it is a better approximation of the ancestral synapsid sacrum than in *O. retroversus*, because the second sacral rib makes contact with the ilium in the former species. There are no described ophiacodontid specimens with three sacral vertebrae [16], [20].

**Figure 6:**
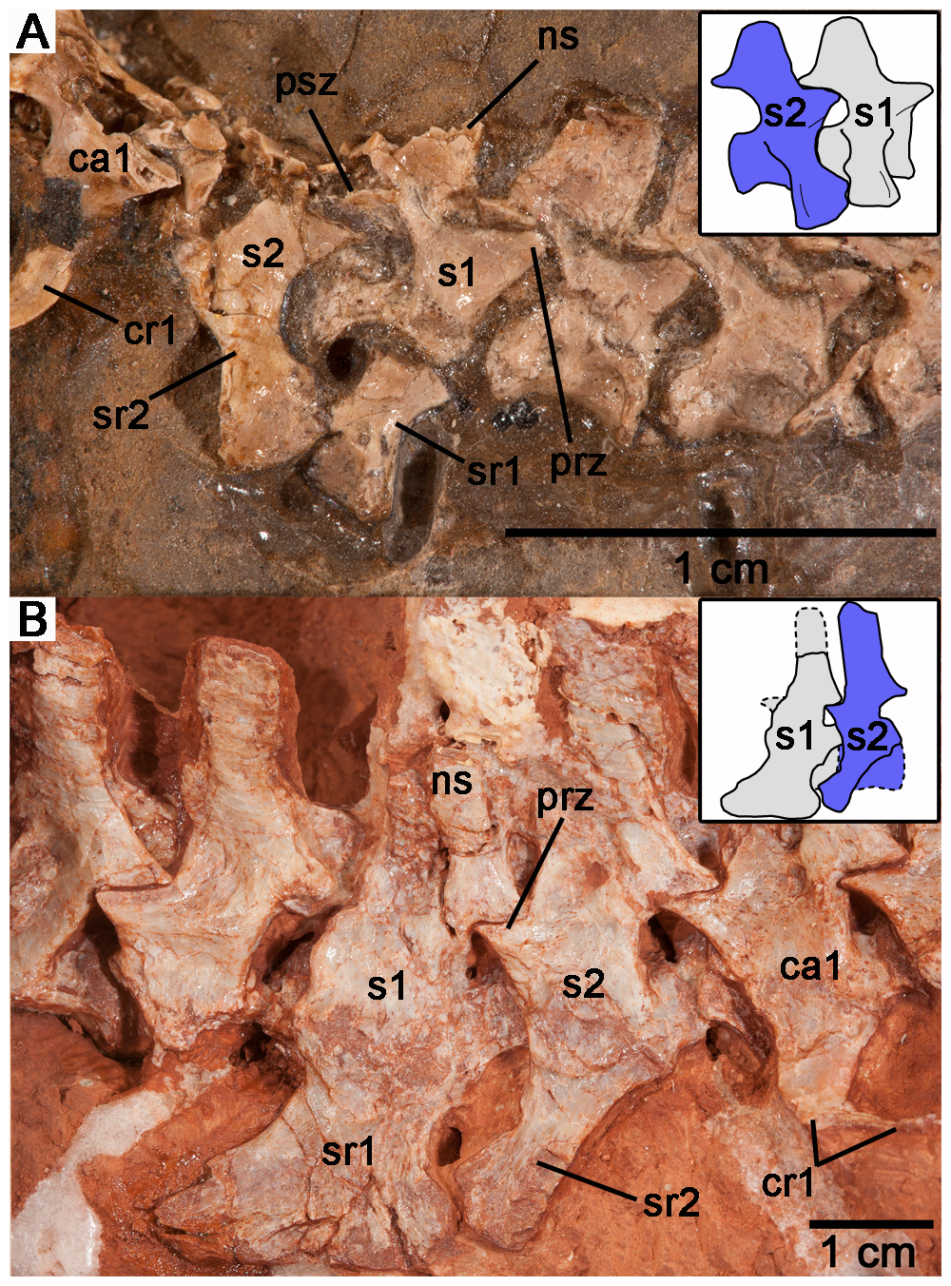
Sacral regions of the basal varanopid *Archaeovenator hamiltonensis* (KUVP 12483) and the varanodontine *Varanodon agilis* (FMNH UR 986). A: Sacral region of KUVP 12483 and reconstruction of the sacral vertebrae (inset). B: Sacral region of FMNH UR 986 and reconstruction of the sacral vertebrae (inset). *Abbreviations*: ca1, first caudal vertebra; cr1, first caudal rib; ns, neural spine; s1–2, sacral vertebrae 1–2; sr1–2, sacral ribs 1–2; prz, prezygapophysis of first sacral vertebra; psz, postzygapophysis of first sacral vertebra.

Varanopids were agile predators that persisted from the Late Carboniferous to the Middle Permian [21], [22]. Although Williston [10] described the derived varanopid *Varanops* as having three sacral vertebrae, Campione and Reisz [13] suggested that all varanopids retained two sacrals. The geologically and phylogenetically oldest varanopid known, *Archaeovenator hamiltonensis* (KUVP 12483) possesses two sacral vertebrae (Figs. 5E, 6A). The distal ends of the sacral ribs are sutured together and form a continuous, cupped articulation with the ilium [21]. The two pairs of sacral ribs are also fused to their respective centra. *A. hamiltonensis* is unusual among early synapsids in that the anterior sacral rib is slightly smaller than the posterior rib, whereas all other early synapsids have significantly larger anterior sacral ribs (Fig. 5). More derived varanopids, such as the large varanodontine *Varanodon agilis* (FMNH UR 986) from the Middle Permian of Oklahoma, possesses only two sacrals (Figs. 5F, 6B), but the Lower Permian varanopid *Aerosaurus wellesi* clearly possesses a small third pair of sacral ribs [23]. In *A. wellesi* the first sacral ribs are the largest and have the broadest distal expansions, which overlap the distal portions of the second pair of sacral ribs. The second pair of ribs is smaller, and the third pair consists of tiny projections, much thinner and shorter than the anterior two pairs and would have barely reached the posteromedial surface of the iliac blade [23]. The diminutive size of the posteriormost sacral ribs suggests that that they are modified caudal ribs. These descriptions indicate that some varanopids did indeed possess a third pair of sacral ribs, but that the ancestral condition for Varanopidae is to possess only two.

Edaphosaurids were large herbivorous synapsids that were ecological equivalents to caseids [24] and persisted from the Late Carboniferous to the Early Permian in North America [7], [8], [18]. Early edaphosaurids may have had only two sacral vertebrae, based on descriptions of fragmentary material of *Ianthasaurus hardestiorum* [25]. Unfortunately, only the first sacral rib is preserved in one specimen of *I. hardestiorum* from the Royal Ontario Museum in Toronto, Canada (ROM 29940), but its greatly expanded distal end and the morphology of the ilium suggested to Reisz and Berman [25] that there was only room for the first sacral rib and a small second sacral rib to form the sacroiliac joint. Another edaphosaurid, *Lupeosaurus kayi* has a partial sacral series, but does not show confidently whether it had two or three sacrals [19]. By comparison, three sacrals are present in a specimen of *Edaphosaurus* from the Museum of Comparative Zoology at Harvard University, Cambridge, Massachusetts (MCZ 1372). Unfortunately, the authors were unable to examine the sacral vertebrae of articulated specimens first hand, and must rely on a mounted cast of a complete *Edaphosaurus boanerges* (ROM 7985) and the following description of a mounted skeleton (MCZ 1372) from Romer and Price ([8]: 385, 392):

“Two sacrals have been generally thought to have been present, but purely on negative evidence. However, a third is definitely seen in a specimen of *E. boanerges* (MCZ 1372). The sacral ribs are apparently unfused distally. Each ends in a flattened surface facing almost directly outwards and is leaf-shaped, broad above and narrowing below. The first two sacrals are moderately large; the third, in the only known instance, is much smaller and has only a tiny terminal plate.”

“[MCZ 1372] shows in addition to two large sacral ribs a third and smaller rib which turned forward to articulate with the ilium.”

Given the paucity of postcranial remains of basal edaphosaurids, it is difficult to determine how the configuration of the sacrum differs between *Ianthasaurus* and *Edaphosaurus* and thus makes it difficult to state if an anterior caudal vertebra was incorporated into the ancestral two-vertebra sacrum or if it was a posterior dorsal vertebra incorporated anteriorly. Nevertheless, the described morphology of the sacral ribs in the mounted skeleton (MCZ 1372) and the cast (ROM 7985) of *E. boanerges* bears resemblance to the sacral series in *Casea broilii*, where the third sacral vertebra is the smallest and reaches anteriorly to make a small contact with the ilium [8] (Fig. 5G). It is likely that, similar to caseids and the varanopid *Aerosaurus wellesi*, the third sacral vertebra in *Edaphosaurus* was added posteriorly and is a modified first caudal vertebra.

Sphenacodontids were a successful group of carnivorous synapsids that extended from the Late Carboniferous to the Early Permian of North America and Europe [8], [22]. Many taxa, including the well-known predators *Dimetrodon* and *Sphenacodon*, have well-described postcrania. From these descriptions, it is clear that all sphenacodontids with preserved sacral regions had three sacral vertebrae [26]–[29] (Fig. 5H). To that end, some specimens of *Dimetrodon* show a tendency toward fusion of adjacent vertebral centra [8]. The first pair of sacral ribs is the largest of the series and the sacral ribs decrease in size posteriorly [8]. As in *Casea broilii*, the distally expanded ends of the three pairs of sacral ribs contact each other, forming a continuous plane of contact with the ilium. Unfortunately, the ancestral condition for Sphenacodontia (haptodontine-grade sphenacodontians, sphenacodontids, and therapsids) in terms of the number of sacrals is difficult to determine, because of a lack of preserved postcrania for several taxa. The sacral anatomy is unknown for the basal taxon *Palaeohatteria* [29], [30], but *Haptodus baylei* possessed three sacral vertebrae [31]. Without postcranial data for most of the early sphenacodontians, it is not clear when and in which taxa the transition from two to three sacral vertebrae took place. Given the proposed phylogenetic positions of the Sphenacodontia within Synapsida [18], they probably independently evolved three sacral vertebrae, and at present, it is most parsimonious to assume a single origin of three sacral vertebrae in Sphenacodontia. According to Benson [18], Edaphosauridae and Sphenacodontia are sister taxa. If the ancestral condition for edaphosaurids is two sacral vertebrae, then sphenacodontians would have independently evolved the three-vertebra sacrum. Interestingly, all therapsids possessed at least three sacral vertebrae [32]–[34]. The earliest therapsids with described postcrania, such as *Biarmosuchus*, had three sacral vertebrae and this appears to have been the ancestral condition for Therapsida [35]. Therapsids presumably inherited the three-sacral condition from the pelycosaurian-grade sphenacodontians, and do not show a reversal to two sacrals [33], [36].

**Figure 7:**
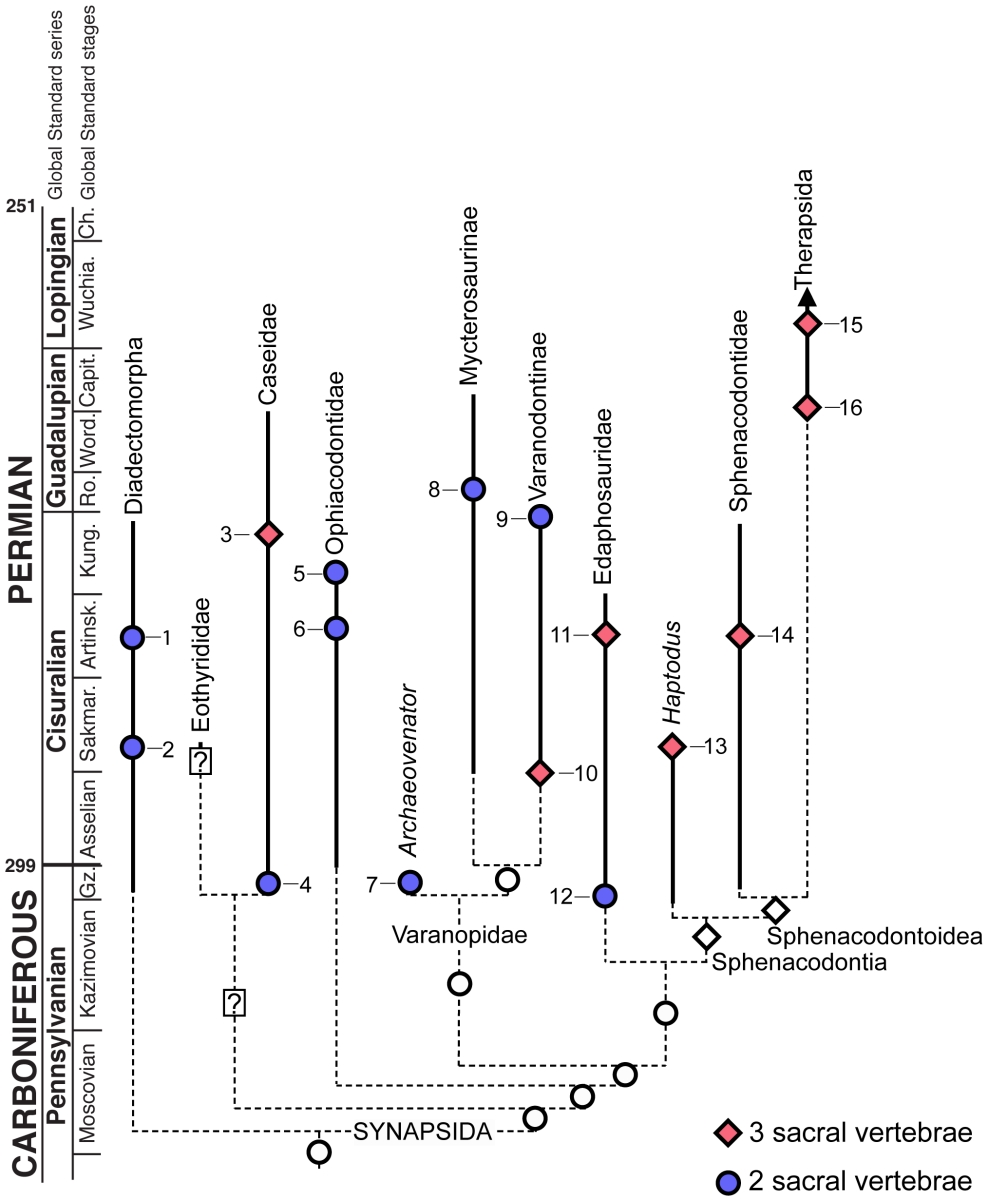
The distribution of sacral vertebral counts mapped on a time-calibrated phylogeny of basal synapsids (modified from Sues and Reisz [42], Laurin and Reisz [41], Fröbisch et al. [43], and Campione and Reisz [13]). 1, *Diadectes sp.*; 2, *Orobates pabsti*; 3, *Casea broilii*; 4, *Eocasea martini*; 5, *Varanosaurus acutirostris*; 6, *Ophiacodon retroversus*; 7, *Archaeovenator hamiltonensis*; 8, *Heleosaurus scholtzi*; 9, *Varanodon agilis*; 10, *Aerosaurus wellesi*; 11, *Edaphosaurus boanerges*; 12, *Ianthosaurus hardestiorum*; 13, *Haptodus baylei*; 14, *Dimetrodon limbatus*; 15, *Biarmosuchus antecessor*; 16, *Suminia getmanovi*. Solid lines indicate known stratigraphic distributions of taxa, dashed lines indicate ghost lineages. White circles and diamonds correspond to hypothetical sacral counts for clades based on observed sacral counts of representative taxa. Stratigraphic occurrences and descriptions of the sacral regions for these taxa were taken from [5], [6], [8], [13], [19], [21], [23], [25], [34], [35], [44]–[47].

To date, no attempt has been made to document sacral evolution in basal synapsids. Whereas our study highlighting convergent evolution of three sacral vertebrae is by no means an exhaustive survey, it provides a basis for hypothesizing the convergent acquisition of three sacral vertebrae in caseids, varanopids, edaphosaurids, and possibly sphenacodontians (Fig. 7). These basal synapsids range from the Late Carboniferous to the Middle Permian, with some lineages attaining massive body sizes [5], [8]. To that end, some caseids and edaphosaurids attained body masses approaching or greatly exceeding 100 kg [5], [37]. It would be tempting to conclude that the evolution of an additional sacral vertebra in these groups is related to such dramatic increases in body size, but as previously mentioned, the earliest occurrence of three sacrals in caseids occurs in small-bodied forms. Furthermore, some ophiacodontids attained large sizes, with *Ophiacodon major* attaining lengths of approximately three meters [18] and yet no ophiacodontid had more than two sacral vertebrae [16]. Müller et al. [2] posited that variations in vertebral numbers in major amniote clades may be less related to body size and more to locomotion and habit. The development of the third sacral rib attachment to the pelvis in synapsids may support this hypothesis. The only osseous contact between the appendicular and axial skeletons in tetrapods occurs between sacral ribs and the ilium, such that any major modifications to the connection between the pelvic girdle and the vertebral column would be reflected in sacral morphology. Unlike mammals, the sacroiliac joint in most basal synapsids was accomplished primarily by connective tissue between the sacral ribs and the ilia and these two elements of the sacral region were not fused [9], [10]. As such, the addition of a third sacral rib would add more stability to this connection and more effectively transfer the forces exerted by the hind limb and pelvic girdle to the axial skeleton. As has been shown here, the independent transitions from two to three sacral vertebrae in non-therapsid synapsids most likely took place during the Early Permian (Fig. 7). Once established, the three-vertebra sacrum became the stable condition for several synapsid groups, including caseids, edaphosaurids, and sphenacodontians.

### Is the Third Sacral Vertebra in Synapsids a Posterior Dorsal or an Anterior Caudal?

Without developmental data it is impossible to say with certainty if all of these early synapsid taxa incorporated a posterior dorsal or an anterior caudal vertebra into the ancestral two-vertebra sacral region. Nevertheless, the observed pattern in the fossil record indicates that separate homeotic shifts in the positions of the dorsal-sacral or sacral-caudal boundaries occurred in multiple lineages of Paleozoic synapsids (Fig. 7). The question remains whether there is osteological evidence that this shift occurred cranially or caudally in these groups. In dinosaurs, there has been extensive discussion on whether or not some groups added vertebrae to the sacrum anteriorly, posteriorly, or both [38]. The primordial first and second sacrals in sauropods, for example, are marked by specific features, such as central fusion and the peculiar shapes of the sacral ribs, which are found even in taxa with several additional sacrals, making it easy to distinguish between the addition of presacral and caudal vertebrae [39]. It appears that early sauropods added a posterior dorsal and an anterior caudal vertebra to the sacral region [39]. Unfortunately very few landmarks exist in Paleozoic synapsid sacral vertebrae to shed light on the same question. Nevertheless, three anatomical observations can be drawn from the synapsid material presented here and in the literature: (1) the earliest members of each synapsid clade that possess three sacral vertebra consistently have a large anterior, a smaller second, and an even smaller third pair of sacral ribs [8], [23]; (2) in Paleozoic synapsids with two or three sacrals the distal end of the anteriormost sacral rib is cupped posteriorly by the second sacral rib in lateral view (the anterior surface of the second sacral rib is concave and articulates with the anteriormost sacral rib); and (3) in nearly all Paleozoic amniotes with two or three sacrals, the anteriormost sacral vertebra has widely separated prezygapophyses and much more narrow postzygapophyses [17], [40].

It is more parsimonious to hypothesize that the anteroposterior size gradient of the three sacral ribs was the result of the addition of a smaller caudal rib to the posterior end of the region. Indeed, the only varanopid with three sacral vertebrae may be important in this regard, because the third pair of sacral ribs in *Aerosaurus wellesi* is extremely small and reaches anteriorly to barely make contact with the ilium and the second pair of sacral ribs [23]. Conversely, this taxon would have to have gained a large sacral rib attachment anteriorly and drastically reduced the size of the posteriormost sacral rib. Furthermore, the concave anterior surface of the second sacral rib in ophiacodontids [9], [19] and varanopids (Figs. 5, 6) may well correspond to the same concavities along the second sacral ribs in *Dimetrodon* [8] (Fig. 5H) and *Casea broilii* (Figs. 1, 3). The second sacral rib in basal amniotes serves mainly as a brace for the first, extending anteriorly and cupping the distal end of the first sacral rib [9]. In sphenacodontids and caseids with three sacral ribs the anterior surface of the third sacral rib is convex and seems to serve simply as an extension of the sacroiliac contact. The anatomy of the sacral ribs in *Edaphosaurus* is extrapolated from the literature (Fig. 5G), but it would be interesting to see if the topological relationships of the three sacral ribs resemble those of caseids and synapsids in the future.

## Conclusions

Unlike previous descriptions of the sacral series of the type genus for Caseidae [6], [10], this new specimen of *Casea broilii* provides a three-dimensional view of the sacrum and shows that it is a well-integrated unit, where the distal ends of all three sacral ribs contact each other and form a single continuous plane of contact with the ilium. Interestingly, when compared to the earliest caseid *Eocasea martini*, it seems that most of the morphological features that characterize caseid sacral morphology evolved in early, small-bodied caseids and were conserved for most of the evolutionary history of the clade.

Similar trends toward expansion of the sacral series have also occurred in other early synapsids, but this is the first comparative review of sacral anatomy across Paleozoic synapsids. This is primarily due to the paucity of descriptions of Paleozoic synapsid posctcrania. The traditional view was that synapsids ancestrally had two pairs of sacral ribs attached to the ilia [41]. Descriptions of basal members of Varanopidae, Caseidae, and Edaphosauridae support this view [5], [21], [25], but this also indicates that these groups probably independently evolved three sacral vertebrae (Fig. 7). From our review of the literature and of the sacral anatomy in caseids it is clear that in all Paleozoic synapsids with three sacral vertebrae, the serial variation in size in both the vertebrae and the ribs is consistent. The anterior sacral ribs are consistently the largest, with the most extensive contacts with the ilia, and the posterior sacral ribs are the smallest (Fig. 5). As suggested by Carrier [1] selective pressures for weight-bearing or more efficient locomotory styles and increasingly terrestrial lifestyles may have promoted the repeated acquisition of three sacral vertebrae in Synapsida. Furthermore all the available evidence supports the hypothesis that the addition of a third vertebra to the sacrum occurred from the caudal series in early synapsids, with the first caudal being incorporated into the sacrum.
